# Assessment of Patient Safety Culture Among Healthcare Professionals in Oman: A Cross-Sectional Study

**DOI:** 10.1155/tswj/7398293

**Published:** 2025-03-06

**Authors:** Zainab M. Al-Zadjali, Heba Ibrahim Awadh, Moon Fai Chan, Sulaiman Dawood Al Sabei, Qamra S. Al-Sariri, Ruhina Aimaq, Phiona Gimono, Yahya M. Al-Farsi

**Affiliations:** ^1^Department of Family Medicine and Public Health, College of Medicine and Health Sciences, Sultan Qaboos University Muscat, Muscat, Oman; ^2^Directorate General of Quality Assurance Center, Ministry of Health, Muscat, Oman; ^3^Fundamentals and Nursing Administration Department, College of Nursing, Sultan Qaboos University, Muscat, Oman

**Keywords:** healthcare professionals, Oman, patient safety culture

## Abstract

**Background:** Patient safety (PS) is a worldwide concern affecting countries at all health system stages. Three million people die each year worldwide due to medical errors and unsafe care. Medical malpractice cases have increased in the Sultanate of Oman, although the reasons for this increase are poorly understood, and there are not many studies on PS.

**Aim:** This study is aimed at assessing PS culture among healthcare professionals in Oman's healthcare facilities.

**Methods:** This cross-sectional study used a national PS culture database maintained by the Directorate General of Quality Assurance at the Ministry of Health. The data was collected using a validated hospital survey on PS culture tool with Cronbach's alpha of 0.87 in the English version which was distributed online to 1599 full-time healthcare professionals in Oman; the response rate was 99%. A stratified random sampling technique was used. The study examined the relationship between items using *t*-tests, chi-squared tests, regression, and odds ratio.

**Results:** Out of the 1599 healthcare professionals who participated in the study, 16 were excluded and only 1583 healthcare professionals were included; the majority 842 (53.2%) were working in nonprimary healthcare (non-PHC). The global average proportion of reported adverse events' positive response rates (PRRs) was significantly higher in the PHC group compared to the non-PHC group (50.0% vs. 47.6%) (*p* < 0.04). Staffing (OR 1.55; 95% CI [1.24–1.93]), teamwork across units (OR 1.37; 95% CI [1.07–1.75]), and organizational learning (OR 1.26; 95% CI [1.02–1.57]) were significantly higher than other domains. The female group showed significantly higher PRR in “staffing” (OR 1.27; 95% CI [1.00–1.62]) (*p* < 0.05). Similarly, older age demonstrated higher PRR in “nonpunitive response to errors” (OR 1.28; 95% CI [1.05–1.57]) (*p* < 0.02), the nursing profession exhibited higher PRR in “communication openness” (OR 1.57; 95% CI [1.24–1.98]) (*p* < 0.001), and advanced work experience was significantly higher in “management support” (OR 1.30; 95% CI [1.07–1.60]) (*p* < 0.01).

**Conclusion:** The study reports that primary healthcare professionals in Omani healthcare institutions have higher PRRs in critical PSC domains like teamwork, supervisor expectations, organizational learning, and staffing compared to non-PHC professionals. They also scored highest in communication openness and management support. The study suggests interventions focusing on staffing adequacy, teamwork, and communication strategies can enhance PS culture among healthcare professionals.

## 1. Introduction

Globally, patient safety (PS) is a major public health concern. Three million people die each year worldwide due to medical errors and unsafe care [1]. According to the WHO [1], up to 4 out of every 100 hospitalized patients in low- to middle-income countries die due to adverse events related to healthcare. Worldwide, common sources of patient harm include unsafe injection at 16 billion per year, patient falls occur on 3–5 per 1000 bed-days, patient misidentification accounts for 12.3%, pressure ulcers affect more than 1 in 10 adults, medication errors happen at 1 in 30, and diagnostic errors occur at 5%–20%, and yet over 50% of the harm is preventable [1]. Additionally, adverse events affect 10% of patients in hospitals, leading to increased costs, injuries, disabilities, and mortality [2].

Several studies have been conducted in Oman on PS; one of the study reported an increase in medical malpractice cases particularly in Muscat Governorate's public healthcare facilities; to be specific, 20.1% occurred in obstetrics and gynecology, 19.7% in internal medicine, 17.6% in surgery, and 13.8% in orthopedics; these are the most affected specialties; however, the specific factors driving this increase are not well documented [3]. Another study reported that the safety culture in maternity units was below the desired standard [4]. And nurses have a bad attitude toward PS [5]. Several studies conducted in the GCCs (Gulf Cooperation Countries) reported that patient safety culture (PSC) varied from poor to moderate and the common problems were observed in staffing and nonpunitive response to errors [6, 7].

PSC is a key metric that is tracked by every healthcare institution worldwide to improve quality of care and PS [8]. Positive PSC is linked to reduced medication errors, improved infection control, shorter hospital stays, and lower mortality rates [1, 9, 10]. Nevertheless, medical malpractice can increase healthcare costs, including extended hospital stays, additional treatments, and litigation costs [1, 9, 10]. According to WHO, PS is a global priority and called for the implementation of PS programs in all healthcare settings [11]. The Directorate General of Quality Assurance Center under the Ministry of Health (MoH) in Sultanate of Oman embraced the WHO initiative for PS and has done tremendous work in achieving safe healthcare. In 2015, the MoH adopted the Patient Safety Friendly Hospital Initiative (PSFHI), which has been implemented in 10 MoH hospitals and 3 private hospitals with a target to implement in all public and private hospitals [11–14]. Despite all these interventions, cases of malpractice and medical errors in the Sultanate of Oman show an upward trend, and several studies have not fully explored PSC [3, 8, 15].

Evaluating PSC among healthcare professionals is essential, as it provides insights into existing challenges and areas for improvement. A positive safety culture is closely linked to reduced medical errors, better patient outcomes, and an overall enhancement in the quality of care. This study sought to assess PSC among healthcare professionals in Oman's healthcare facilities, offering evidence to inform targeted interventions.

## 2. Materials and Methods

### 2.1. Study Design

The study was conducted as a cross-sectional study, as the primary data collection was completed by the MoH from December 2021 to February 2022.

This cross-sectional study used a national PSC database maintained by the Directorate General of Quality Assurance at the MoH. The database was created using a validated hospital survey on PSC tool distributed online to 1599 full-time healthcare professionals in Oman.

### 2.2. Sample and Sample Size

The sample size was 1599 healthcare professionals from the national database of PSC at the MoH; 16 of them were excluded due to failure to meet the inclusion criteria. The response rate was 99%. The healthcare professions included doctors, nurses, dentists, pharmacists, radiographers, laboratory technicians, and administrative and ancillary staff.

### 2.3. Sampling Method

A systematic random sampling method was used in each stratum, selecting 10% of healthcare professionals from each stratum to ensure a representative sample [16]. The 10% threshold was determined based on a balance between logistical feasibility and statistical representativeness. Given the large pool of healthcare professionals in Oman, this proportion allowed for adequate coverage across various professional groups while maintaining a manageable dataset for analysis. Furthermore, prior studies on PSC have used similar proportions to achieve robust statistical power and generalizability [8].

### 2.4. Data Collection Tools

The primary data was collected by the MoH using an online questionnaire, the *Hospital Survey on Patient Safety Culture (HSOPSC) Version 2.0*, developed by the Agency for Healthcare Research and Quality (AHRQ) in 2016 to assess PSC among healthcare professionals in different settings. The HSOPSC was selected due to its comprehensive structure, strong psychometric properties, and widespread global use in healthcare settings, which facilitates cross-study comparisons and benchmarking. Compared to other tools, such as the Safety Attitudes Questionnaire (SAQ) or the Manchester Patient Safety Framework (MaPSaF), the HSOPSC provides a broader scope, assessing 12 key dimensions of PSC, including teamwork within units, supervisor/manager expectations and actions promoting PS, organizational learning–continuous improvement, management support for PS, overall perceptions of PS, feedback and communication about error, communication openness, nonpunitive response to errors, teamwork across units, staffing, handoffs and transitions, and frequency of events reported. The tool's multidimensional approach allows for a thorough assessment of both individual and organizational factors influencing PSC. Furthermore, the HSOPSC's validated reliability (Cronbach's alpha 0.87) and adaptability across different healthcare settings make it a robust choice for evaluating PSC in Oman.

The original English version of the HSOPSC was utilized for this study. The decision to use the English version was based on the professional environment in Oman, where English is commonly used in healthcare settings, especially for documentation and communication among healthcare professionals. Furthermore, most healthcare professionals in Oman receive their education and training in English, ensuring their proficiency and comfort in responding to the survey accurately [17]. The MoH has previously validated the use of this tool in its original language in similar studies, confirming its relevance and comprehensibility in the local context [8].

In this study, we utilized secondary data collected by the MoH from December 2021 to February 2022 using the *HSOPSC Version 2.0*. While the data collection was performed by the MoH, the primary data analysis including data cleaning, statistical testing, and interpretation was conducted by the authors of this study.

The HSOPSC is a valid and reliable tool developed based on previous literature, cognitive tests, and factor analysis [8, 18] The construct validity of the questionnaire showed a significant correlation (*r* > 0.7), indicating the scale's validity. Additionally, the overall Cronbach's alpha of 0.87 for the English version demonstrates the questionnaire's reliability [19]. The instrument comprises 42 items grouped into 12 measures, along with two questions on overall grade for PS and reported events in the past 12 months. The scale used a 5-point Likert scale which ranges from “*strongly disagree*” to “*strongly agree*” or from “*never*” to “*always*” when relevant. A global safety grade between “poor” and “excellent” and the number of reported incidents in the past 12 months were also assessed [19].

### 2.5. Inclusion and Exclusion Criteria

Healthcare professionals working full time were included in the study, and those absent on duty were excluded.

### 2.6. Data Analysis

The data was analyzed using SPSS (Version 23.0, IBM). The HSOPSC tool comprised positively and negatively worded items scored on a 5-point Likert scale. The percentage of positive, negative, and neutral responses for each composite was calculated, and composites with at least 70% positive responses were considered strengths, while those scoring lower were identified as areas for improvement. Demographic characteristics were analyzed using descriptive statistics, and Student's *t*-test was used to examine potential statistically significant differences in the survey questions.

The chi-squared test examined significant differences between categorical items. Univariate logistic regression modeled the association between selected predictors and each PS domain. In addition, odds ratios (ORs) and 95% confidence interval analyses were used. A *p* value of 0.05 or less was considered statistically significant.

### 2.7. Ethics and Ethical Approval

Ethical approval for this study was obtained from the Research and Ethical Review and Approval Committee at the Centre of Research and Studies in the MoH, ID: MoH/CSR/22/26596. The study adhered to the Declaration of Helsinki's ethical framework. The study was conducted using the approved national PSC database. Participants were sent an online consent form, emphasizing confidentiality and voluntary participation, with the ability to withdraw at any time. The survey was anonymous and confidential.

## 3. Results

Table 1 displays the sociodemographic details of participants based on their healthcare affiliation. Out of 1583 healthcare professionals, 842 (53.2%) worked at nonprimary healthcare (non-PHC) facilities, while 741 (46.8%) worked at primary healthcare (PHC) facilities. Most of them were females (77.4%) and over half were aged 31–40 (55.5%). The majority (98.0%) were affiliated with the MoH than the 2.0% in private health facilities. Many healthcare professionals (58.5%) were from nursing backgrounds, 23.6% from a medical background, and others from various roles; interestingly, 32.5% had over 5 years of experience. Furthermore, there were statistically significant differences in gender distribution (*p* < 0.001), work experience (*p* < 0.02), professional background (*p* < 0.04), and qualifications (*p* < 0.01) between healthcare professionals working in PHC and non-PHC facilities.

Table 2 shows selected attitude indices toward PSC in healthcare institutions by healthcare-level affiliation. The majority (91.2%) reported direct patient interaction, with a significantly higher proportion in the PHC group (93.7% vs. 89.1%) (*p* < 0.001). Additionally, most (39.0%) reported good PS practices. The pattern was consistent across the two groups, with non-PHC and PHC both at 39.5% and 38.3%, respectively. However, the PHC group reported a significantly higher proportion of fair ratings than the non-PHC group (16.3% vs. 3.9%) (*p* < 0.01). About half of the participants reported nonadverse events, with no statistically significant difference between the PHC and non-PHC groups (*p* > 0.12).

Table 3 shows the distribution of response rates across PSC domains, stratified by healthcare-level affiliation. In the PHC group, the positive response rate (PRR) was less than 50% in 12 out of 29 items, accounting for 41.3%. The PRR ranged from 8.4% to 87.5%. The highest PRR was for “busy help” (87.5%), followed by “management top priority” (85.2%). The lowest PRR was for “afraid to ask” (8.4%), followed by “problem happens” (17.2%). In the non-PHC group, the PRR was less than 50% in 13 out of 29 items, accounting for 44.8%. The PRR ranged from 12.6% to 90.1%, with statistically significant differences observed between the PHC and non-PHC groups in 11 of the 29 items (*p* < 0.05).

**Figure 1:**
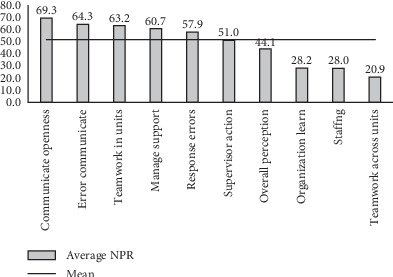
Average PRR across different domains of patient safety culture among all respondents (*N* = 1583). NPR, number of positive response.

Figure 1 shows the average PRRs across different PSC domains for all respondents in the PHC and non-PHC groups. The global average PRR for the PHC group was significantly higher than that for the non-PHC group (50.0% vs. 47.6%) (*p* < 0.04). Additionally, significant differences in mean PRR were observed in the “supervisor expectation and action” (*p* < 0.002), “organizational learning” (*p* < 0.003), and “staffing” (*p* < 0.028) domains.

Table 4 displays the analysis of healthcare-level affiliation and each domain of PSC. The PHC group had a significantly higher PRR compared to non-PHC across most domains, including staffing (OR 1.55; 95% CI [1.24–1.93]), “teamwork across units” (OR 1.37; 95% CI [1.07–1.75]), and “organizational learning” (OR 1.26; 95% CI [1.02–1.57]).

Table 5 displays the univariate regression modeling of the association between gender and each domain of PSC. The female group had a significantly higher PRR than males in the “staffing” domain (OR 1.27; 95% CI [1.00–1.62]) (*p* < 0.05) and a significantly lower PRR in the “supervisor expectation and action” domain (OR 0.74; 95% CI [0.55–0.99]) (*p* < 0.05).

Table 6 presents the univariate logistic regression modeling of the association between age and each domain of PSC. The older age group had a higher PRR in some domains, with “nonpunitive response to errors” significantly higher among the older age (OR 1.28; 95% CI [1.05–1.57]) (*p* < 0.02), while the lowest PRR was observed in the “teamwork across units” domain (OR 0.64; 95% CI [0.54–0.77]) (*p* < 0.001).

Table 7 shows the univariate logistic regression modeling of the association between professional background (nursing) and each domain of PSC. The nursing profession group had a significantly higher PRR than the nonnursing professionals across some domains. “Communication openness” domain (OR 1.57; 95% CI [1.24–1.98]) (*p* < 0.001) was significantly higher, followed by “management support” and “supervisor expectations and actions.” Also, PRR was significantly lower in most domains such as “staffing” (OR 0.48; 95% CI [0.39–0.59]) (*p* < 0.001), followed by “communication about errors” and “teamwork across units.”

Table 8 displays the univariate logistic regression modeling for the association between work experience and each domain of PSC. It revealed a significant association between advanced work experience and higher prevalence rate ratio in the “management support” domain (OR 1.30; 95% CI [1.07–1.60]) (*p* < 0.01).

## 4. Discussion

PS is vital in healthcare, and a strong PSC is crucial to prevent harm due to unsafe healthcare practices. This study assessed PSC among healthcare professionals in Oman, comparing PHC (health centers, polyclinics) and non-PHC facilities (tertiary care like hospitals) [20]. The PHC in Oman is considered as the first line in receiving and managing patients with mild and moderate conditions; however, it refers severe case to tertiary hospitals (non-PHC) due to the structure wise and availability of more specialization health professional. The study findings on attitude reported that PHC professionals interact directly with patients thus reflecting PHC's continuous patient contact [8, 21–23]. Furthermore, the PHC group reported a significantly higher proportion of fair ratings compared to the non-PHC group. This could be due to PHC settings handling a broader spectrum of health conditions and patients with complex needs, which may present greater challenges to PS [22].

Regarding response rates across different domains of PSC, the results show that PRR was less than 50% in several items of PSC among all respondents, indicating a low PSC among the respondents. The lowest PRR is for the items “afraid to ask” (8.4%) and “problems happen” (17.2%) indicating a lack of psychological safety among healthcare providers. These findings underscore the need to enhance aspects of PSC like communication and nonpunitive responses to errors. This could be contributed by challenges related to hierarchical organizational culture or trust issues among team members, potentially hindering effective communication and collaboration [24].

Enhancing psychological safety is critical for creating an environment where healthcare providers feel comfortable voicing concerns and contributing to improving PS [8, 21, 25, 26]. In addition, other studies' findings reported that communication openness is key for PSC [27, 28]. Furthermore, the highest PRR was observed for the items “busy help” (87.6%) and “effective team” (84.4%), indicating a positive PSC in these areas. Previous studies reported that effective teamwork and communication are essential in ensuring PS and preventing medical errors [10, 29, 30]. Encouraging positive aspects of PSC can significantly impact overall care quality. The PHC group reported lower readiness in 11 of 29 items, indicating a need to focus more on PSC. The global average readiness for all respondents was 51.6%, showing a moderate level of PSC. The “communication openness” domain showed the highest mean PRR, whereas “teamwork across units” reported the lowest PRR. These findings are consistent with the previous studies that indicate a positive association between communication openness and PSC [2, 27].

The PHC group had a higher PRR than the non-PHC group in most of the PSC domains, with significant differences in 11 items. Both groups achieved the highest scores in *communication openness* and *management support*. Additionally, the PHC group displayed a higher PRR in the domains of *teamwork within units*, *supervisor expectations and actions*, *organizational learning*, and *staffing*, indicating a positive impact of affiliation with PHC.

Several factors could explain the differences observed between PHC and non-PHC groups. PHC settings typically emphasize team-based, patient-centered care due to their role in providing comprehensive and continuous care across various health conditions. This close-knit, multidisciplinary collaboration fosters stronger teamwork within units and more effective communication among healthcare professionals. Additionally, PHC facilities often have flatter hierarchical structures compared to tertiary hospitals, facilitating more approachable leadership and clear supervisor expectations, which could explain the higher PRR in *supervisor expectations and actions*.

Furthermore, PHC settings are often the first point of contact for patients, requiring healthcare professionals to engage in continuous learning to manage a wide spectrum of health conditions. This necessity for ongoing professional development may contribute to the higher PRR observed in the *organizational learning* domain. In contrast, non-PHC (tertiary) settings may face more complex administrative structures, heavier workloads, and more specialized roles, which could hinder teamwork and reduce perceived management support. These structural differences may account for the lower PRR in non-PHC groups in several domains.

The univariate categorical data analysis reported significant associations between PHC affiliation and higher PRR across most of the domains, with the strongest associations observed in the domains of “staffing,” “teamwork across units,” and “organizational learning.” These findings suggest that healthcare-level affiliation may be essential in promoting PSC in healthcare settings. Moreover, female participants had a higher PRR than male participants in most domains, particularly in the staffing domain. Females exhibited better safety awareness than males likely due to their caring nature [31]. These findings were consistent with previous studies that suggested that women tend to have better PS outcomes than men [31, 32]. Nevertheless, female gender was associated with a lower PRR in the “supervisor expectation and action” domain, while older age (41 or above) was linked to higher PRR in several domains. These findings align with previous research indicating a positive association between age and perceptions of PSC [31]. This finding suggests that there may be a need to examine the role of gender in leadership and management practices in healthcare [31]. The lowest association was observed in the “teamwork across units” domain, followed by “communication about errors” and “organizational learning.” One possible explanation for this finding is that older HCWs may have less exposure to teamwork across units, as they may have been practicing in a specific unit for a long time. This may lead to reduced unit communication and collaboration and lower overall teamwork perceptions [33].

The study reported that older healthcare workers have a stronger association with the nonpunitive response to errors, indicating their greater comfort with this concept and their willingness to report incidents and errors, which is crucial for PSC [34]. The nursing profession group had a significantly higher PRR than the nonnursing professionals across some domains. These findings support previous studies highlighting the significant role of healthcare professionals' backgrounds in shaping PSC within healthcare organizations, especially in nursing [35, 36]. Lastly, advanced work experience was associated with a lower PRR in several domains, although statistically nonsignificant except in management support. This suggests that work experience alone may not be sufficient to promote a positive safety culture, and other factors, such as training and education, may also be essential.

### 4.1. Limitations

It is important to acknowledge that this study has limitations, including the use of a cross-sectional design, reliance on self-reported data, potential influence on data quality by the MoH, and limited participation of management-level respondents.

## 5. Conclusion

This study provides valuable insights into PSC among healthcare professionals in Omani healthcare institutions. The findings revealed that PHC professionals demonstrated higher PRRs in several critical PSC domains, including *teamwork within units*, *supervisor expectations and actions*, *organizational learning*, and *staffing*, compared to non-PHC (tertiary) professionals. Both PHC and non-PHC groups scored highest in *communication openness* and *management support*, indicating these as strengths across healthcare settings.

The results also highlighted significant associations between demographic factors and PSC, with female healthcare professionals reporting higher PRR in *staffing* and older professionals showing greater comfort in *nonpunitive response to errors*. These insights suggest that targeted interventions focusing on staffing adequacy, teamwork, and communication strategies, particularly in non-PHC settings, can enhance overall PSC.

Strengthening PSC through leadership engagement, continuous organizational learning, and supportive management practices should be prioritized by policymakers and healthcare leaders to improve the quality and safety of care in Oman.

## Figures and Tables

**Table 1:** Sociodemographic characteristics of participants, stratified by healthcare level, Oman, 2022.

**Characteristics**	**Total**	**PHC**	**Non-PHC**	
	**(** **N** = 1583**)**	**(** **n** = 741**)**	**(** **n** = 842**)**	
	**n** ** (%)**	**n** ** (%)**	**n** ** (%)**	**p** ** value**
*Gender*				0.001⁣^∗^
Female	1226 (77.4)	604 (81.5)	622 (73.9)	
Male	357 (22.6)	137 (18.5)	220 (26.1)	
*Age (years)*				0.61
20–39	195 (12.3)	101 (13.6)	94 (11.2)	
31–40	878 (55.5)	453 (61.1)	425 (50.5)	
41–50	412 (26.0)	157 (21.2)	255 (30.3)	
51–60	87 (5.5)	28 (3.8)	59 (7.0)	
61 or above	11 (0.7)	2 (0.3)	9 (1.1)	
*Institute type*				0.48
Ministry of Health	1551 (98.0)	728 (98.2)	823 (97.7)	
Private institute	32 (2.0)	13 (1.8)	19 (2.3)	
*Professional background*				0.04⁣^∗^
Nursing	931 (58.5)	429 (57.9)	502 (69.6)	
Medical^a^	375 (23.7)	164 (22.1)	211 (25.1)	
Other clinical position	192 (12.1)	97 (13.1)	95 (11.3)	
Administrative/support	85 (5.4)	51 (6.9)	34 (4.0)	
*Work experience (years)*				0.02⁣^∗^
Less than 1	121 (7.6)	48 (6.5)	73 (8.7)	
1–5	421 (26.6)	227 (30.6)	194 (23.0)	
6–10	513 (32.5)	256 (34.5)	257 (30.5)	
*Qualifications*				0.01⁣^∗^
High school	34 (2.1)	31 (4.2)	3 (0.4)	
Diploma	696 (44.0)	364 (49.1)	332 (39.4)	
Bachelors	584 (36.9)	268 (36.2)	316 (37.5)	
Masters	170 (10.7)	39 (5.3)	131 (15.6)	
PhD	38 (2.4)	16 (2.2)	22 (2.6)	
MD	41 (2.6)	21 (2.8)	20 (2.4)	
Fellowship	20 (1.3)	2 (0.3)	18 (2.1)	

Abbreviations: MD, doctor of medicine; PHC, primary healthcare.

^a^Medical includes dentists.

⁣^∗^Significant.

**Table 2:** Selected indices of attitude toward patient safety culture within the healthcare institution, stratified by healthcare-level affiliation, Oman, 2022.

**Characteristics**	**Total**	**PHC**	**Non-PHC**	
	**(** **N** = 1583**)**	**(** **n** = 741**)**	**(** **n** = 842**)**	
	**n** ** (%)**	**n** ** (%)**	**n** ** (%)**	**p** ** value**
*Direct interaction*				0.001⁣^∗^
Yes	1444 (91.2)	694 (93.7)	750 (89.1)	
No	139 (8.80)	47 (6.3)	92 (10.9)	
*Patient safety rating*				0.01⁣^∗^
Poor	33 (2.1)	2 (0.3)	31 (3.7)	
Fair	154 (9.7)	121 (16.3)	33 (3.9)	
Good	617 (39.0)	284 (38.3)	333 (39.5)	
Very good	535 (33.8)	202 (27.3)	333 (39.5)	
Excellent	244 (15.4)	132 (17.8)	112 (13.3)	
*Adverse event reporting*				0.12
None	805 (50.9)	379 (51.1)	426 (50.6)	
1–2	460 (29.1)	206 (27.8)	254 (30.2)	
3–5	199 (12.6)	94 (12.7)	105 (12.5)	
6–10	58 (3.7)	28 (3.8)	30 (3.6)	
11 or more	61 (3.9)	34 (4.6)	27 (3.2)	

Abbreviation: PHC, primary healthcare.

⁣^∗^Significant.

**Table 3:** Response rates across different domains of patient safety culture, stratified by healthcare-level affiliation.

**Domain**	**PHC**				**Non-PHC**				**p** ** value**
	**(** **n** = 741**)**				**(** **n** = 842**)**				
	**N**	**NPR**	**NOR**	**PRR**	**N**	**NPR**	**NOR**	**PRR**	
*Teamwork within units*									
AQ1 Effective team	737	573	164	77.7	842	759	83	90.1	0.001
AQ3 Longer hours	733	479	254	65.3	823	288	535	35.0	0.001
AQ4 Review process	738	552	186	74.8	829	626	203	75.5	0.743
AQ11 Rushed negative	722	216	506	29.9	821	244	577	29.7	0.933
BQ1 Manager serious	734	584	150	79.6	832	627	205	75.4	0.047
*Supervisor expectation and action*									
BQ2 Manager shortcuts	731	165	566	22.6	822	199	623	24.2	0.447
BQ3 Manager actions	727	562	165	77.3	822	654	168	79.6	0.28
*Organizational learning*									
AQ6 Mistakes against	709	227	482	32.0	803	231	572	28.8	0.17
AQ13 Lack support	726	214	512	29.5	810	186	624	23.0	0.004
*Management support*									
FQ1 Manage top priority	737	628	109	85.2	835	688	147	82.4	0.131
FQ3 Manage interested	722	180	542	24.9	822	226	596	27.5	0.271
FQ2 Manage resources	731	531	200	72.6	833	598	235	71.8	0.708
*Overall perceptions*									
AQ10 Learn and no blame	734	473	261	64.4	824	507	317	61.5	0.248
EQ1 Department rating	741	334	407	45.1	842	445	397	52.9	0.002
FQ5 Shift left out/handover	688	161	527	23.4	772	133	639	17.2	0.004
*Communication about error*									
CQ1 Informed errors	734	532	202	72.5	827	495	332	59.9	0.001
CQ3 Informed changes	725	487	238	67.2	828	564	264	68.1	0.692
CQ5 Authority speak	711	429	282	60.3	796	466	330	58.5	0.479
*Communication openness*									
CQ2 Discuss prevent	734	561	173	76.4	836	642	194	76.8	0.865
CQ4 Staff speak	730	507	223	69.5	824	574	250	69.7	0.929
CQ6 Authority open	701	420	281	59.9	787	498	289	63.3	0.183
*Nonpunitive response to errors*									
AQ8 Help during a busy time	738	646	92	87.5	839	736	103	87.7	0.909
AQ12 Changes evaluated	725	547	178	75.4	825	615	210	74.5	0.682
CQ7 Afraid to ask	722	61	661	8.4	817	108	709	13.2	0.003
*Teamwork across units*									
FQ4 Information left out	699	165	534	23.6	771	142	629	18.4	0.015
*Staffing*									
AQ2 Enough staff	741	375	366	50.6	836	238	598	28.5	0.001
AQ5 Temporary staff	685	177	508	25.8	771	182	589	23.6	0.324
AQ7 Person written up	705	260	445	36.9	791	246	545	31.1	0.018
AQ14 Problems happen	719	124	595	17.2	811	102	709	12.6	0.01

Abbreviations: NOR, number of other response; NPR, number of positive response; PHC, primary healthcare; PRR, positive response rate.

**Table 4:** Univariate logistic categorical data analysis of the association of healthcare-level affiliation and each domain of patient safety culture.

**Domain**	**PHC (** **n** = 741**)**		**Non-PHC (** **n** = 842**)**		**OR**	**95% CI**		**p** ** value**
	**NPR** ^**a**^	**%**	**NPR**	**%**		**Lower**	**Upper**	
Teamwork within units	485	65.5	514	61.1	1.21	0.98	1.48	0.07
Supervisor expectation and action	256	49.9	328	51.9	0.92	0.76	1.13	0.43
Organizational learning	370	30.7	437	25.9	1.26	1.02	1.57	0.04⁣^∗^
Management support	371	60.9	405	60.6	1.01	0.83	1.24	0.91
Overall perceptions	227	44.3	218	43.9	1.01	0.83	1.24	0.90
Communication about error	514	66.7	624	62.2	1.21	0.99	1.49	0.07
Communication openness	451	68.6	510	69.9	0.94	0.76	1.16	0.55
Nonpunitive response to errors	290	57.1	332	58.5	0.94	0.77	1.15	0.56
Teamwork across units	328	23.6	370	18.4	1.37	1.07	1.75	0.01⁣^∗^
Staffing	413	32.6	472	23.9	1.55	1.24	1.93	0.001⁣^∗^
Global average	494	50.0	524	47.6	1.10	0.90	1.34	0.35

Abbreviations: CI, confidence interval; OR, odds ratio; PHC, primary healthcare.

^a^Percentages for each domain represent the proportion of positive responses (NPR) out of the total number of respondents in each group. For example, for teamwork within units in the PHC group, the percentage is calculated as 485/741 × 100 = 65.5%. This method was applied consistently across all domains.

⁣^∗^Significant.

**Table 5:** Univariate logistic regression modeling of the association between gender and each domain of patient safety culture.

**Domain**	**Female gender**	**95% CI**		
	**OR**	**Lower**	**Upper**	**p** ** value**
Teamwork within units	1.37	0.97	1.94	0.08
Supervisor expectation and action	0.74	0.55	0.99	0.05⁣^∗^
Organizational learning	0.97	0.74	1.26	0.08
Management support	0.85	0.62	1.16	0.31
Overall perceptions	1.14	0.89	1.46	0.30
Communication about error	1.16	0.90	1.49	0.26
Communication openness	1.06	0.80	1.40	0.70
Nonpunitive response to errors	1.14	0.79	1.66	0.48
Teamwork across units	1.04	0.77	1.42	0.78
Staffing	1.27	1.00	1.62	0.05⁣^∗^
Global average	1.07	0.80	1.44	

Abbreviations: CI, confidence interval; OR, odds ratio; PHC, primary healthcare.

⁣^∗^Significant.

**Table 6:** Univariate logistic regression modeling of the association between age (41 years or above) and each domain of patient safety culture.

**Domain**	**Older age**	**95% CI**		
	**OR**	**Lower**	**Upper**	**p** ** value**
Teamwork within units	1.24	1.03	1.49	0.02⁣^∗^
Supervisor expectation and action	0.83	0.71	0.97	0.02⁣^∗^
Organizational learning	0.78	0.67	0.90	0.001⁣^∗^
Management support	1.17	0.98	1.40	0.09
Overall perceptions	1.24	1.08	1.42	0.001⁣^∗^
Communication about error	0.78	0.69	0.90	0.001⁣^∗^
Communication openness	1.13	0.97	1.32	0.11
Nonpunitive response to errors	1.28	1.05	1.57	0.02⁣^∗^
Teamwork across units	0.64	0.54	0.77	0.001⁣^∗^
Staffing	0.99	0.86	1.12	0.83
Global average	1.01	0.86	1.19	

Abbreviations: CI, confidence interval; OR, odds ratio; PHC, primary healthcare.

⁣^∗^Significant.

**Table 7:** Univariate logistic regression modeling of the association between professional background (nursing) and each domain of patient safety culture.

**Domain**	**Nursing profession**	**95% CI**		
	**OR**	**Lower**	**Upper**	**p** ** value**
Teamwork within units	0.83	0.62	1.09	0.18
Supervisor expectation and action	1.32	1.04	1.69	0.02⁣^∗^
Organizational learning	0.98	0.78	1.22	0.85
Management support	1.41	1.08	1.85	0.01⁣^∗^
Overall perceptions	0.78	0.63	0.97	0.02⁣^∗^
Communication about error	0.58	0.47	0.72	0.001⁣^∗^
Communication openness	1.57	1.24	1.98	0.001⁣^∗^
Nonpunitive response to errors	1.02	0.75	1.39	0.89
Teamwork across units	0.71	0.55	0.91	0.01⁣^∗^
Staffing	0.48	0.39	0.59	0.001⁣^∗^
Global average	0.97	0.76	1.24	

Abbreviations: CI, confidence interval; OR, odds ratio; PHC, primary healthcare.

⁣^∗^Significant.

**Table 8:** Univariate logistic regression modeling of the association between work experience (6–10 years) and each domain of patient safety culture.

**Domain**	**Advanced experience**	**95% CI**		
	**OR**	**Lower**	**Upper**	**p** ** value**
Teamwork within units	1.08	0.82	1.42	0.59
Supervisor expectation and action	0.85	0.67	1.08	0.19
Organizational learning	0.96	0.77	1.21	0.75
Management support	1.30	1.07	1.60	0.01⁣^∗^
Overall perceptions	1.22	0.99	1.51	0.06
Communication about error	0.89	0.72	1.10	0.28
Communication openness	1.05	0.83	1.33	0.70
Nonpunitive response to errors	0.98	0.72	1.33	0.91
Teamwork across units	0.94	0.73	1.22	0.65
Staffing	1.06	0.86	1.30	0.58
Global average	1.03	0.82	1.31	

Abbreviations: CI, confidence interval; OR, odds ratio; PHC, primary healthcare.

⁣^∗^Significant.

## Data Availability

Data is available on request through direct contact of the Professor: Yahya M. Al-Farsi, Department of Family Medicine and Public Health, College of Medicine and Health Sciences, Sultan Qaboos University; email: ymfarsi@squ.edu.om.

## Nomenclature

AHRQ: Agency for Healthcare Research and Quality
GCCs: Gulf Cooperation Countries
HSOPSC: Hospital Survey on Patient Safety Culture
MoH: Ministry of Health
PHC: primary healthcare
PRRs: positive response rates
PSC: patient safety culture
PS: patient safety
